# Strigolactone insensitivity affects the hormonal homeostasis in barley

**DOI:** 10.1038/s41598-025-94430-2

**Published:** 2025-03-18

**Authors:** Magdalena Korek, Devang Mehta, Glen R. Uhrig, Agata Daszkowska-Golec, Ondrej Novak, Weronika Buchcik, Marek Marzec

**Affiliations:** 1 Institute of Biology, Biotechnology and Environmental Protection, Faculty of Natural Sciences, University of Silesia in Katowice, Jagiellonska 28, 40-032 Katowice, Poland; 2 Department of Biological Sciences, University of Alberta, 11455 Saskatchewan Drive, Edmonton, AB T6G 2E9 Canada; 3 Laboratory of Growth Regulators, Faculty of Science, Palacký University and Institute of Experimental Botany, The Czech Academy of Sciences, Olomouc, Czech Republic

**Keywords:** *Hordeum vulgare*, Branching, Strigolactones, Phytohormone cross-talk, Biotechnology, Plant sciences

## Abstract

In response to environmental changes, plants continuously make architectural changes in order to optimize their growth and development. The regulation of plant branching, influenced by environmental conditions and affecting hormone balance and gene expression, is crucial for agronomic purposes due to its direct correlation with yield. Strigolactones (SL), the youngest class of phytohormones, function to shape the architecture of plants by inhibiting axillary outgrowth. Barley plants harboring the mutation in the *HvDWARF14* (*HvD14*) gene, which encodes the SL-specific receptor, produce almost twice as many tillers as wild-type (WT) Sebastian plants. Here, through hormone profiling and comparison of transcriptomic and proteomic changes between 2- and 4-week-old plants of WT and *hvd14* genotypes, we elucidate a regulatory mechanism that might affect the tillering of SL-insensitive plants. The analysis showed statistically significant increased cytokinin content and decreased auxin and abscisic acid content in ‘bushy’ *hvd14* compared to WT, which aligns with the commonly known actions of these hormones regarding branching regulation. Further, transcriptomic and proteomic analysis revealed a set of differentially expressed genes (DEG) and abundant proteins (DAP), among which 11.6% and 14.6% were associated with phytohormone-related processes, respectively. Bioinformatics analyses then identified a series of potential SL-dependent transcription factors (TF), which may control the differences observed in the *hvd14* transcriptome and proteome. Comparison to available *Arabidopsis thaliana* data implicates a sub-selection of these TF as being involved in the transduction of SL signal in both monocotyledonous and dicotyledonous plants.

**Supplementary Information:**

The online version contains supplementary material available at 10.1038/s41598-025-94430-2.

## Introduction

Strigolactones (SL) represent a class of plant hormones regulating various aspects of plant growth and development, including inhibiting shoot branching through intricate interactions with other hormonal pathways^1^. However, the detailed SL-related mechanism that shapes the plants’ architecture, a crucial agronomic trait directly affecting the plants’ yield, is still unravelled. The initial identified downstream genes, whose expression is SL-dependent, encode proteins belonging to the TEOSINTE BRANCHED1/CYCLOIDEA/PROLIFERATING CELL FACTOR1 (TPC) family^2^. The most extensively documented member of this family in the literature is the BRANCHED1 (BRC1), which acts as a transcription factor (TF) locally in buds and regulates the shoot branching by inhibiting the axillary bud outgrowth. The Arabidopsis (*Arabidopsis thaliana*) study showed that *atbrc1* mutants display a ‘bushy’ phenotype, which can not be rescued by SL application^3^. Moreover, SL-insensitive and SL-depleted plants exhibited a notable decrease in the accumulation of *BRC1* transcripts^3–8^. It was shown that the expression of *BRC1* is constitutively up-regulated in plants deficient in SL-repressor proteins, while its expression is downregulated in gain-of-function SL-repressor mutants^9,10^. However, no experimental data shows that *BRC1* is a direct target of SL-repressor. Contrary, both genetic and physical interactions between rice (*Oryza sativa*) SQUAMOSA PROMOTER BINDING PROTEIN-LIKE14 (OsSPL14) and TEOSINE BRANCHED1 (OsTB1), a rice BRC1 orthologue, have been described, leading to the hypothesis that *OsTB1* transcription is regulated by OsSPL14, known in literature as a negative regulator of branching^11^. Further studies confirmed the direct interaction between SL repressor and OsSPL14, recognising SL as a key phytohormone that profoundly influences shoot architecture^11^. However, this complex regulatory network governing shoot branching also involves dynamic interactions between SL and other plant hormones, specifically auxins (AUX) and cytokinins (CK), orchestrating a finely tuned regulatory system.

The pivotal role of AUX in regulating shoot branching was first discovered in 1930s, when experiments showed that removing the shoot apex in plants triggered the activation and growth of axillary buds^12,13^. Conversely, the treatment of decapitated stumps with AUX suppresses bud outgrowth^14^. The AUX canalisation model assumes that AUX forms narrow transport streams that connect AUX-synthesising tissues with regions where AUX regulates diverse molecular pathways^15^. Polar AUX transport is mediated by PIN-FORMED efflux carrier proteins (PINs), with PIN1 being a crucial protein involved in the transport of AUX within the stem^16^. The phenotype of SL-insensitive or SL-depleted mutants can be explained by SL influence on AUX transport via regulating the expression and polar localization of AUX transporters. Consistent with this idea, rice and Arabidopsis SL mutants have increased AUX transport and PIN1 accumulation^17,18^. At the same time, *rac*-GR24 (a synthetic analogue of SL) can rapidly induce depletion of PIN1 from the plasma membrane of stem xylem parenchyma cells^19,20^. Moreover, expression of genes *MORE AXILLARY GROWTH 3* and *4* (*MAX3* and *4*) encoding SL-biosynthesis enzymes are positively AUX-regulated^21–24^. This suggests that AUX and SL modulate each other’s levels required for the coordinated control of axillary branching. Additionally, *BRC1* is quickly downregulated after decapitation^3,25^, while applying AUX can promote *BRC1* expression in buds^2,3^. These observations highlight cross-talk between AUX and SL in regulating plant architecture.

While SL and AUX act to induce the *BRC1* expression in the buds, an adverse effect on the expression of *BRC1* and its homolog has been observed after CK application. The *BRC1* transcripts levels decreased in a CK dose-dependent manner in rice^26^, pea (*Pisum sativum*)^5^ and *Chrysanthemum*^27^, thus highlighting the antagonistic action of CK versus SL and AUX in shoot branching regulation. Moreover, the Arabidopsis *altered meristem program1* (*amp1*) mutants accumulating higher CK levels showed increased bud outgrowth resulting from reduced *BRC1* expression^28^. In addition, the knock-out of SL-regulated *SPL13* resulted in a higher accumulation of CK and transcripts levels of CK synthesis gene *ISOPENTENYL TRANSFERASES 1* (*IPT1*) in the stem nodes^29^. The result suggests that SL inhibits lateral bud growth by suppressing CK biosynthesis. In parallel, AUX controls local CK biosynthesis in the nodal stem in apical dominance^30^.

Here, using SL-insensitive barley (*Hordeum vulgare*) mutant *hvd14* and its parent cultivar Sebastian, we performed a phytohormone content profiling with transcriptomic and proteomic analyses to understand the role of SL in barley development. Our analyses allowed us to describe the SL interactions with other phytohormones in shaping the barley architecture and revealed a set of TF that might be involved in SL-related regulatory mechanisms. Together, these data enhanced our understanding of SL influence on phytohormone homeostasis during barley tillering.

## Results

### Mutation in SL receptor promotes tillering in barley

**Fig. 1:**
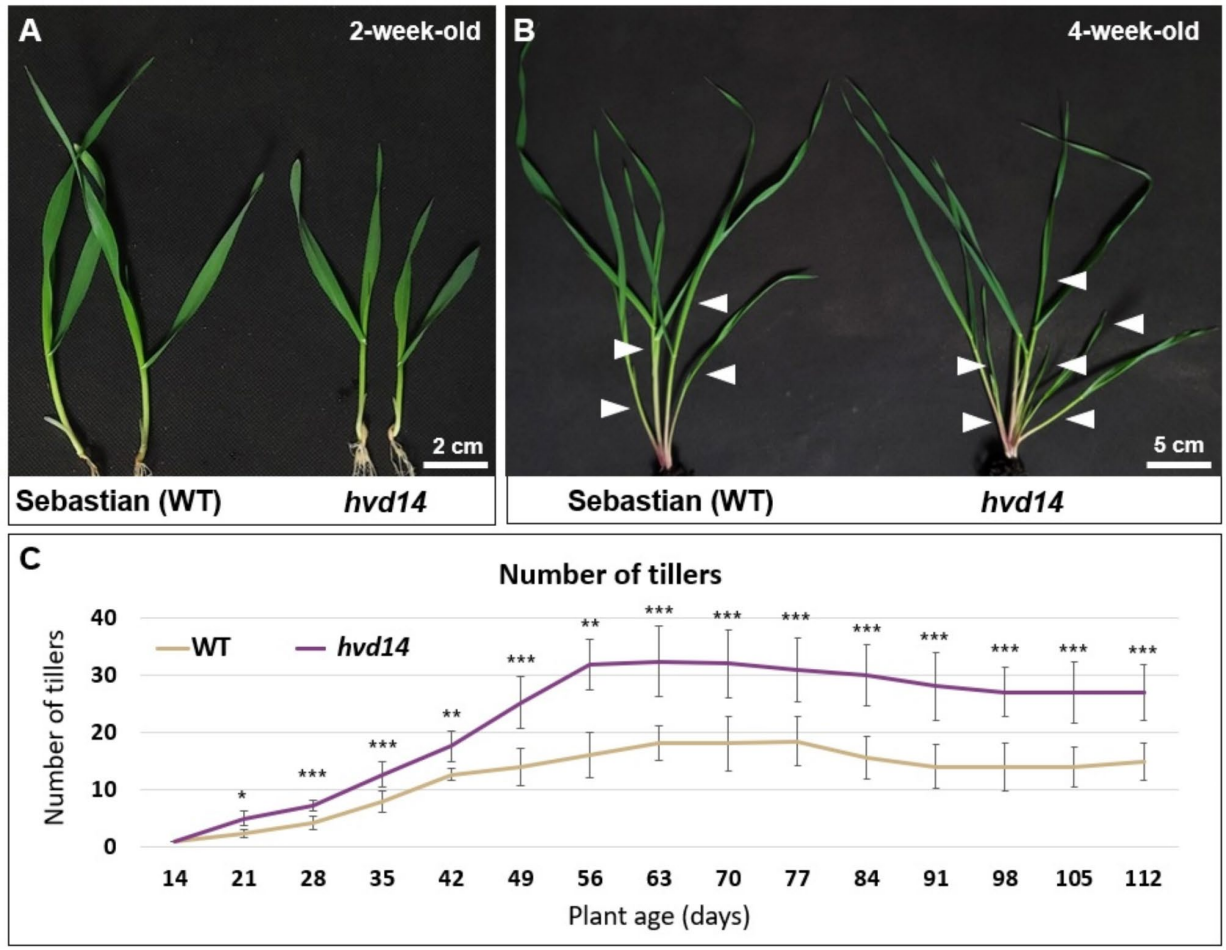
The phenotype of *hvd14*. Shoot architecture of (**A**) 2- and (**B**) 4-week-old Sebastian and *hvd14*. (**C**) Differences in tillers number between WT and mutant plants across 4 months of development. The means ± SE are presented. Asterisks indicate statistically significant differences between genotypes in each time point, as determined by Student’s t-test (*p*-values corresponding *p < 0.05; **p < 0.01; ***p < 0.001).

Barley mutant *hvd14*, carried the single transition (G725A) in the *HvD14* gene (GenBank: KP069479.1), which encodes the SL receptor (HvD14; GenBank: KP069479.1), was identified from a TILLING population^31^. Identified mutation affects the protein structure (G193E) and prevents the binding of hormone molecules, which results in the SL-insensitivity of *hvd14* plants^31^. SL-insensitivity of *hvd14* plants was observed when synthetic analogues of SL, such as *rac*GR24^31^ or GR24^5DS^^32^, were used. Whereas both SL analogues inhibited tillering in the wild-type (WT) Sebastian cultivar, this effect was not observed for *hvd14*^31^. Also, under control conditions, without phytohormonal treatment, a higher number of tillers was produced by *hvd14* compared to WT. Mature *hvd14* plants developed almost twice as many tillers as WT (27 ± 4.9 and 14 ± 3.3, respectively). Differences in shoot architecture become visible and statistically significant in 4-week-old plants (Fig. 1C), and plants in that age were selected for further analysis. Additionally, 2-week-old seedlings of both genotypes before the outgrowth of first tillering tiller buds (Fig. 1A) were included in all experiments.

### Mutation in *HvD14* gene results in altered phytohormone content

**Fig. 2:**
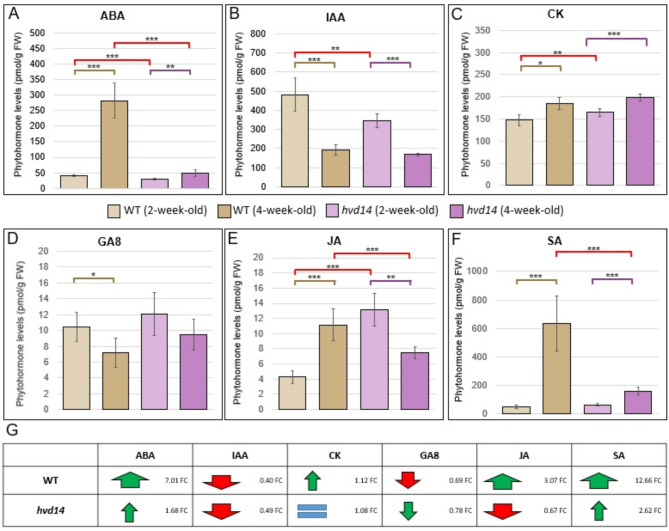
Mutation in *HvD14* alters phytohormone content in barley. Measurement of (**A**) ABA, (**B**) IAA, (**C**) CK, (D) GA8, (**E**) JA, (**F**) SA content of 2- and 4-week-old Sebastian and *hvd14* plants. Asterisks indicate statistically significant differences between samples in Student’s t-test (*p*-values corresponding *p < 0.05; **p < 0.01; ***p < 0.001). (**G**) Changes in the hormonal profiles of WT and the mutant during the early stages of plant development. The red and green arrows represent an increase or decrease in hormone content, respectively, with their size indicating the magnitude of the change. An equal sign denotes no change in the level of the analyzed hormone.

Phytohormones can cooperate, playing antagonistic or synergistic roles, to control different aspects of plant development, with a disturbance in the biosynthesis or signalling pathways of one phytohormone affecting the action of others, manifesting as changes in their content within and/or across plant tissues. Correspondingly, we assessed the phytohormone profiles of WT and *hvd14* plants using 2- and 4-old-week plants (Supplementary Data 1). The content of multiple phytohormones, such as abscisic acid (ABA), indol-3-acetic acid (IAA), brassinosteroids (BR), cytokinins (CK), gibberellins (GAs), jasmonic acid (JA) and salicylic acid (SA), and their intermediates were measured. Only two out of six GAs were detected in barley tissue, GA6 and GA8, and no differences were observed between 2-week-old WT and *hvd14* plants, similar to SA (Fig. 2D,F) (Supplementary Data 1). In the same comparison, WT plants exhibited higher content of ABA (40.32 vs. 28.84 pmol/g FW) and IAA (482.27 vs. 344.69 pmol/g FW) compared to the *hvd14* plants (Fig. 2A,B). However, the opposite results were obtained for CK (147.71 vs. 184.88 pmol/g FW) and JA (4.28 vs. 11.17 pmol/g FW), in which lower content was noticed in WT compared to *hvd14* seedlings (Fig. 2C,E) (Supplementary Data 1). Among all eight BRs, only the 24-nor brassinolide (norBL) was detected in the tissue of 2-week-old seedlings of both genotypes but not in the 4-week-old plants of WT or *hvd14* (Supplementary Data 1). When comparing 4-week-old plants, no statistically significant differences in IAA and GA8 were observed between genotypes, while a significantly higher content of ABA (282.75 vs. 48.48 pmol/g FW), JA (13.16 vs. 7.48 pmol/g FW) and SA (632.62 vs. 159.01 pmol/g FW) was detected in WT comparing to the *hvd14*. Conversely, significantly lower amounts of CK (164.92 vs. 199.32 pmol/g FW), was observed in WT (Fig. 2C) (Supplementary Data 1). The most significant differences in phytohormone content in 2-week-old *hvd14* seedlings were found for ABA (0.72 FC), IAA (0.71 FC) and JA (2.6 FC) compared to WT (Fig. 2A,B,E). Whereas in 4-week-old plants, the most pronounced differences in phytohormone composition were observed for ABA (0.17 FC), SA (0.12 FC) and JA (0.57 FC) comparing WT and *hvd14* (Fig. 2A,E,F) (Supplementary Data 1).

Because the observed differences were related to the plant age, we next assessed changes in phytohormone content related to the plant stage of development within each genotype. Here, a similar pattern of hormonal change was observed for ABA and SA, with higher accumulation found in WT vs. *hvd14*, 7.01 vs. 1.68 FC and 12.66 and 2.62 FC, respectively (Fig. 2G) (Supplementary Data 1). In contrast, IAA content decreased during development in both genotypes at a similar level (0.4 and 0.49 FC in WT and *hvd14*). Opposite trends in phytohormone content were observed for JA, which increased in WT (3.07 FC) and decreased in *hvd14* (0.67 FC), while CK and GA8 increased (1.12 FC) and decreased (0.69 FC), respectively in WT, but did not change or slighty change significantly in *hvd14* (Fig. 2G).

### SL insensitivity affects transcriptome and proteome during barley development

**Fig. 3:**
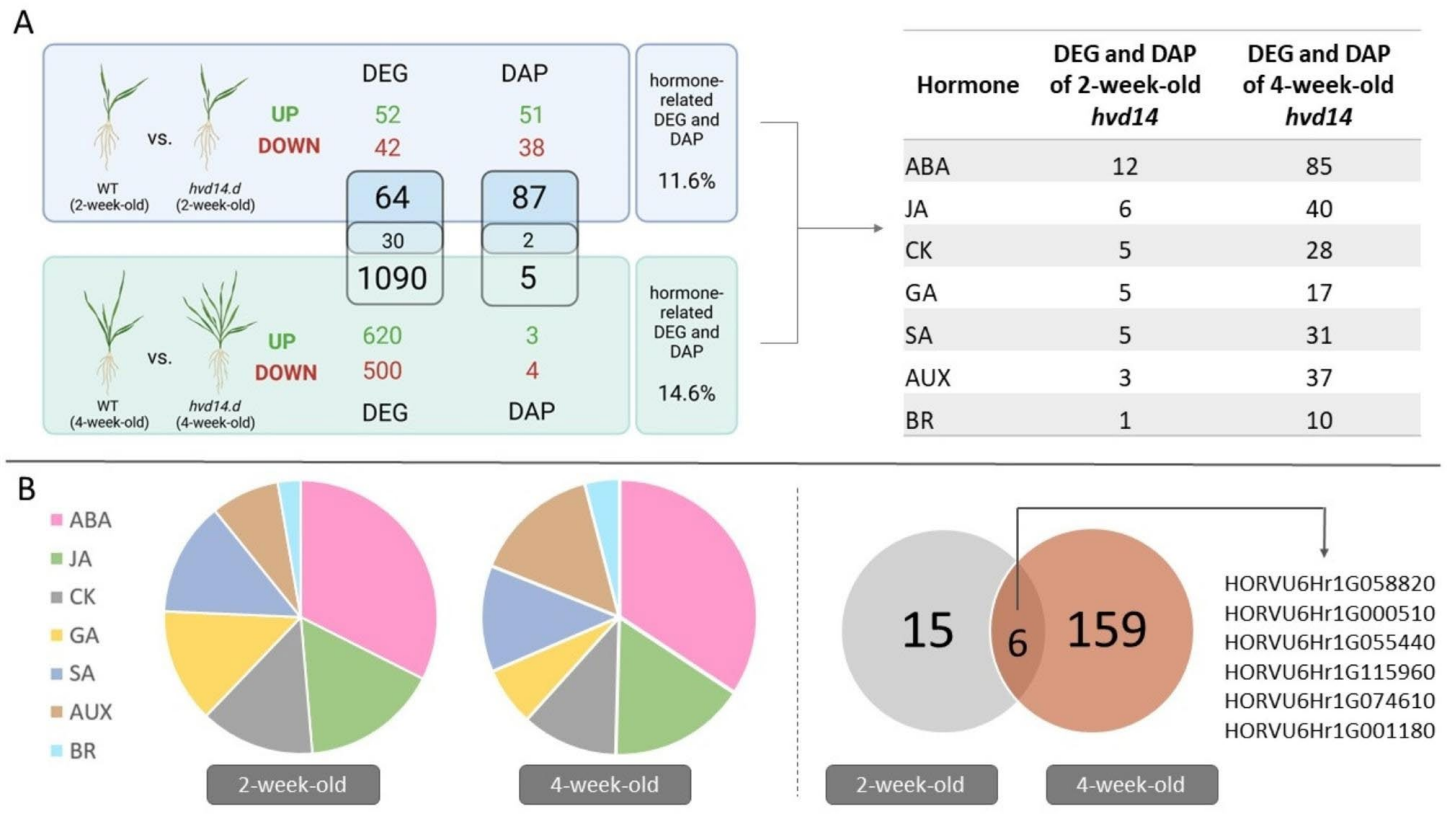
Transcriptome and proteome changes affected by SL-insensitivity. (**A**) The numbers of differentially expressed genes (DEG) and differentially abundant proteins (DAP) identified after the comparison analysis between 2- and 4-week-old Sebastian and *hvd14* plants. The table shows the numbers of DEG and DAP involved in phytohormone-related processes. (**B**) Pie charts showing distribution of hormone-related DEG and DAP of younger and older *hvd14*. Venn diagram shows the numbers of specific and shared hormone-related DEG and DAP (duplicates removed) for 2- and 4-week-old *hvd14* plants. The illustration was created using BioRender (www.biorender.com).

Comparison of WT vs. *hvd14* leaf transcriptomes revealed 94 and 1120 differentially expressed genes (DEG; log2FC ≥ 1 or log2FC ≤ − 1, adjusted *P* value ≤ 0.01) for younger and older plants, respectively (Fig. 3A) (Supplementary Data 2). At both developmental timepoints, a higher number of DEG was up-regulated (54) compared to down-regulated (42) for 2-week-old plants; (620 up- and 500 down-regulated for 4-week-old plants) (Fig. 3A). Among these datasets, most of DEG were specific for either 2-week-old or 4-week-old barley plants, revealing only 30 genes in common. On the other hand, proteome analysis showed the opposite pattern, revealing more differentially abundant proteins (DAP; log2FC ≥ 0.58 (corresponding to a 1.5-fold change) or log2FC ≤ − 0.58, adjusted *P* value ≤ 0.01) for younger plants compared to older ones, which is 89 and 7, respectively (Fig. 3A), with only two DAP in common among the presented contrasting groups.

### Transcriptomic and proteomic analysis reveals phytohormone-associated processes in SL-insensitive barley

The obtained DEG and DAP lists were then used to identify the transcriptome and proteome changes that may affect the mutant’s hormonal balance, to uncover expression regulation mechanisms distributed in *hvd14*. Based on GO terms assigned to identified DEG and DAP, our analysis revealed that 11.6% (21/181) of transcriptomic changes and 14.6% (165/1127) of proteomic changes are associated with phytohormone-related processes for younger and older plants, respectively (Fig. 3A). However, some of the identified genes/proteins were annotated to more than one term linked to phytohormones (Supplementary Data 3). Most DEG and DAP are related to ABA and JA, which aligns with results obtained for phytohormone content measurement, where ABA and JA differences between tested genotypes were the most statistically significant. ABA and JA reflect 12/36 and 6/36 of all GO terms related to phytohormones in 2-week-old pants, as well as 85/248 and 40/248 in 4-week-old plants (Fig. 3A,B). Interestingly, the expression of six hormone-related genes was specifically regulated in the mutant at both time points tested (Fig. 3B, Table 1). Among them are three genes encoding lipoxygenases (LOX), which are associated with the production of three classes of phytohormones: ABA, JA, and SA. Specifically, our *hvd14* mutant showed a decreased content of JA, SA, and ABA, despite the increased expression of the genes encoding LOX during plants growth.

**Table 1:** Phytohormone-related genes with altered expression in comparison of WT vs. *hvd14*, in both 2- and 4-week-old plants.

Hormone	Horvu ID	Gene description (PlantTFDB/UniProt)	2-week old		4-week old	
			log2FC	adj.pval	Log2FC	adj.pval
GA	HORVU6Hr1G058820	PHE ammonia lyase 1	− 0.63	0.0232	− 2.29	8.60E−06
ABA/JA/SA	HORVU6Hr1G000510	lipoxygenase 2	1.75	0.0499	9.04	1.37E−07
CK	HORVU6Hr1G055440	Glycine-rich RNA-binding protein 8	2.71	0.0019	4.45	0.0004
ABA/JA/SA	HORVU2Hr1G115960	PLAT/LH2 domain-containing lipoxygenase family protein	1.48	0.0063	1.65	3.82E−06
ABA/CK	HORVU2Hr1G074610	Histidine kinase 5	3.81	2.42E−06	2.94	1.61E−09
ABA/JA/SA	HORVU5Hr1G001180	lipoxygenase 2	1.14	0.0063	1.57	0.0008

Next, we examined the promoter sequences (1500 bp) of all identified hormone-related DEG and genes encoding DAP to find TF motifs and potential over-arching regulatory TF for both 2- and 4-week-old plants (Supplementary Data 4 & 5). The prepared data allowed us to select TF that recognize binding sites in the promoter sequences of hormone-related DEG and genes encoding DAP, for both 2- and 4-week-old plants. This identified several putative TF that may regulate the expression of genes belonging to all hormone-related categories, including AUX, ABA, JA, GA, BR, CK, and SA. In total, 3 and 29 TF were selected as master regulators of phytohormonal pathways for younger and older plants, respectively (Supplementary Data 5). Interestingly, all 3 TF identified for 2-week-old plants (HORVU5Hr1G113220, HORVU2Hr1G087310, HORVU1Hr1G063610) were also identified in 4-week-old plants. Moreover, 7 TF, including HORVU5Hr1G113220 and HORVU2Hr1G087310, possess a binding site in LOX genes (Supplementary Data 5). Comparison to Arabidopsis homologues of these three genes found they encode OBF-BINDING PROTEIN 3 (OBP3), BABY BOOM (BBM) and PISTILLATA (PI) TF. OBP3 belongs to DNA BINDING WITH ONE FINGER (DOF) TF family, which is involved in a wide range of developmental processes. What is interesting is that the constitutive overexpression of many DOF TF results in plant dwarfing despite the diverse biological functions of these genes in plant growth^33,34^. Additionally, Arabidopsis transgenic lines overexpressing *OBP3* present altered root development and small, yellowish leaves^35^. Both traits are regulated by many hormone-dependent signalling pathways. However, the *OBP3* increased expression was only proved after AUX and SA treatment^35^. The second identified gene, BBM, is one of the members of the AIL/PLT (AINTEGUMENTA-LIKE/PLETHORA) family encoding TF containing an AP2/ERF domain^36^. The presence of AIL/PLT family proteins can be observed in dividing tissues or organs, such as roots, shoots and floral meristems, where they ensure the maintenance of the meristematic state of cells^37^. Additionally, analysis of mutant collections showed that AIL/PLT proteins are dose-dependent regulators of root development. The *plt1 plt2 plt3 bbm* Arabidopsis mutant possesses completely inhibited root growth compared to WT^38^. Phenotypic data about described mutants, such as altered root system architecture—a characteristic feature of SL gene mutants—may further support their involvement in SL signaling. Additionally, it was shown that BBM transcriptionally regulates the activity of AUX-biosynthesis genes, thus promoting its accumulation in seedlings^39^. Moreover, the *BBM* overexpression in transgenic lines of Arabidopsis and *Brassica napus* results in spontaneously forming somatic embryos on seedlings without supplementation of exogenous hormones^40^. Since the embryogenic transition involves changes in hormonal homeostasis, BBM may serve as a strong candidate for an SL-related TF that influences the phytohormonal network. The last gene, PI, encodes a homeotic protein, which, together with APETALA 3 (AP3), plays a role in the formation of petals and stamens in angiosperm flowers^41^. Plants exhibiting mutation in *PI* present a male-sterile phenotype. However, the SL-depleted or SL-insensitive plants have not affected flower development, so the identification of PI as an SL-related TF cannot be excluded.

Lastly, among all detected TF that may regulate hormone-related DEG and DAP we endeavoured to sort out those TF that may bind to the most represented promoter binding elements. This allows us to predict 24 TF with over-represented targets in our dataset (Supplementary Data 6). Moreover, we compared them with 29 TF that were identified as a phytohormone master regulators, thus revealing 10 TF that may be considered as a key TF responsible for ‘bushy’ phenotype of *hvd14* due to phytohormones content alterations (Table 2).

**Table 2:** List of TF with over-represented targets in hormone-related DEG and DAP of 2- and 4-week-old *hvd14.*

HORVU ID	Best HIT in Arabidopsis	Protein family	No. of targets in				Description (PlantTFDB/NCBI)
			2-week-old		4-week-old		
			DEG	DAP	DEG	DAP	
HORVU7Hr1G012840 (MLOC_15776)	AT5G42520	BBR-BPC	3	9	146	10	Specifically binds to GA-rich elements present in regulatory sequences of genes involved in developmental processes
HORVU6Hr1G008870 (MLOC_3855)	AT1G72050	C2H2	4	1	304	3	Required for transcription of 5 S rRNA gene
HORVU0Hr1G007050 (MLOC_24530)	AT5G44210	ERF/AP2	3	4	56	5	Protein contains one AP2 domain
HORVU2Hr1G036710 (MLOC_1876)	AT3G45260	C2H2	0	1	31	0	Functions redundantly with JACKDAW to control root development
HORVU4Hr1G070960 (MLOC_60958)	AT2G02080	C2H2	2	1	31	0	Its phosphorylation is induced under salinity stress by MPK6, regulating plant growth adaptation
HORVU5Hr1G023000 (MLOC_51930)	AT3G62420	bZIP	0	0	19	0	Forms heterodimers with group-C bZIP TF to bind to the ACTCAT cis-element of proline dehydrogenase gene
HORVU6Hr1G069190 (MLOC_73724)	AT5G62940	DOF	2	3	90	3	Induces the formation of interfascicular cambium and regulates vascular tissue development
HORVU5Hr1G018020 (MLOC_23884)	AT4G33280	AP2/B3	0	0	25	0	AP2/B3-like transcriptional factor family protein
HORVU6Hr1G017710 (MLOC_63436)	AT4G34590	bZIP	0	0	21	0	Regulates gene expression of enzyme-coding genes involved in amino acid metabolism
HORVU3Hr1G024210 (MLOC_52112)	AT5G11260	bZIP	0	0	17	0	Plays a role in anthocyanin accumulation, binds to the promoter of *ABSCISIC INSENSITIVE 5* (*ABI5*) and regulates its expression

### Bioinformatic approach predicting SL-related TF

To better understand the molecular mechanisms underpinning the differences of WT and *hvd14* plants, we queried all of DEG and DAP data for TF. Based on amino acid sequences of all DEG and DAP, we found 8 (4.4%, 8/181) and 101 (8,9%, 101/1127) TF in younger and older plants, respectively (Supplementary Data 7). Furthermore, for each of the 109 TF, we identified an Arabidopsis ortholog, and compared obtained list with SL-responsive genes reported by Wang et al. 2020^42^. Here, the authors identified 401 potentially SL-responsive genes using ten-day-old Columbia-0 seedlings treated with 5 μM GR24^4DO^^42^. Among them, four orthologous genes were common with our dataset (*HORVU5Hr1G000490*/*AT3G18550, HORVU5Hr1G068110/AT5G67060, HORVU1Hr1G090250/AT1G64380, HORVU2Hr1G028840/AT2G02820*) (Supplementary Data 7). *AT3G18550*, differentially expressed when comparing 4-week-old *hvd14* and Sebastian plants, encodes a BRC1, whose involvement in SL-related regulation of shoot branching was extensively documented, as described above. The identification of BRC1 exclusively in older and not younger plants might explain the differences in shoot phenotype, as 2-week-old barley WT and *hvd14* plants exhibited similar branching level, in contrast to 4-week-old plants. The role of the remaining three TF (*HORVU5Hr1G068110/AT5G67060, HORVU1Hr1G090250/AT1G64380, HORVU2Hr1G028840/AT2G02820*) in the signal transduction pathway has not yet been functionally tested, but the presence of motifs recognized by them in SL-dependent genes in barley and Arabidopsis indicates their significant function in this process. However, it should be emphasized that none of the 4 TF identified in this approach have been previously identified to control hormone-dependent differences in the transcriptome and proteome observed in *hvd14* (Supplementary Data 5 &6).

Further, we again assessed the promoter sequences in terms of identifying TF binding sites and selecting TF with over-represented targets of DEG and DAP describing differences between WT and mutant plants (Supplementary Data 8). This allowed us to predict TF with significantly over-represented targets in DEG and DAP datasets, showing that 70 and 75 TF may control the proteome and transcriptome changes in younger and older plants, respectively. Comparison of these two datasets enabled the selection of 33 common TF (Supplementary Data 9). We also identified 14 TF, which might regulate the expression of DEG and DAP, and at the same time, their abundance was altered by a mutation in the *HvD14* gene (Supplementary Data 9). These genes might be strong candidates as a master SL-responsive TF participating in SL-signal transduction. Lastly, we identified the TF with over-represented targets in promoters of SL-responsive genes reported by Wang et al. 2020^42^ (Supplementary Data 10). This dataset allowed us to select 79 TF that potentially may regulate SL-responsive genes in the Arabidopsis genome.

**Fig. 4:**
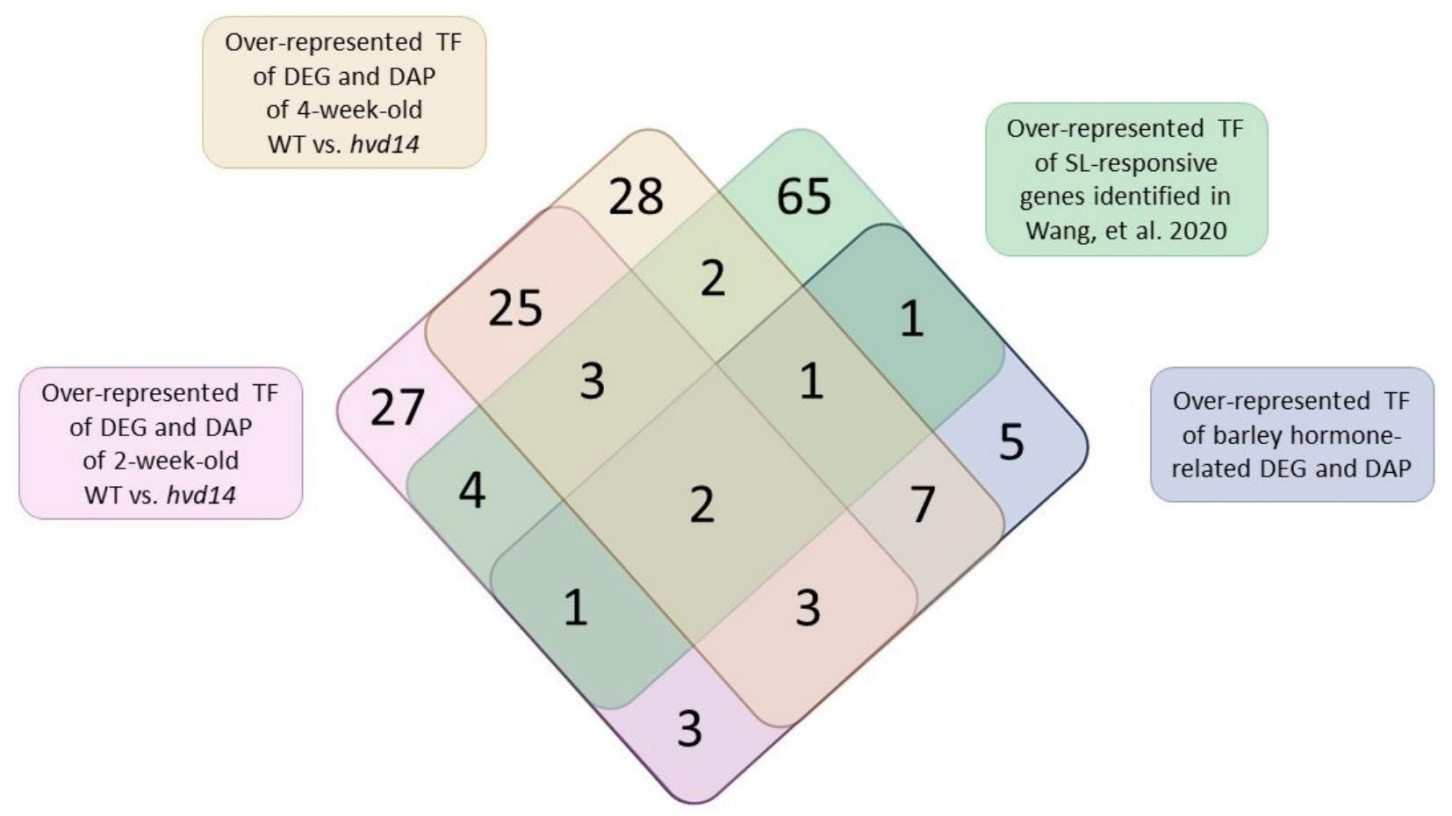
TF with over-represented targets in SL-responsive genes of barley and Arabidopsis. Venn diagram showing numbers of identified SL-responsive TF specific for 2- and 4-week old WT vs. *hvd14* and Arabidopsis SL-responsive genes selected by Wang et al. 2020. The illustration was created using BioRender (www.biorender.com).

**Fig. 5:**
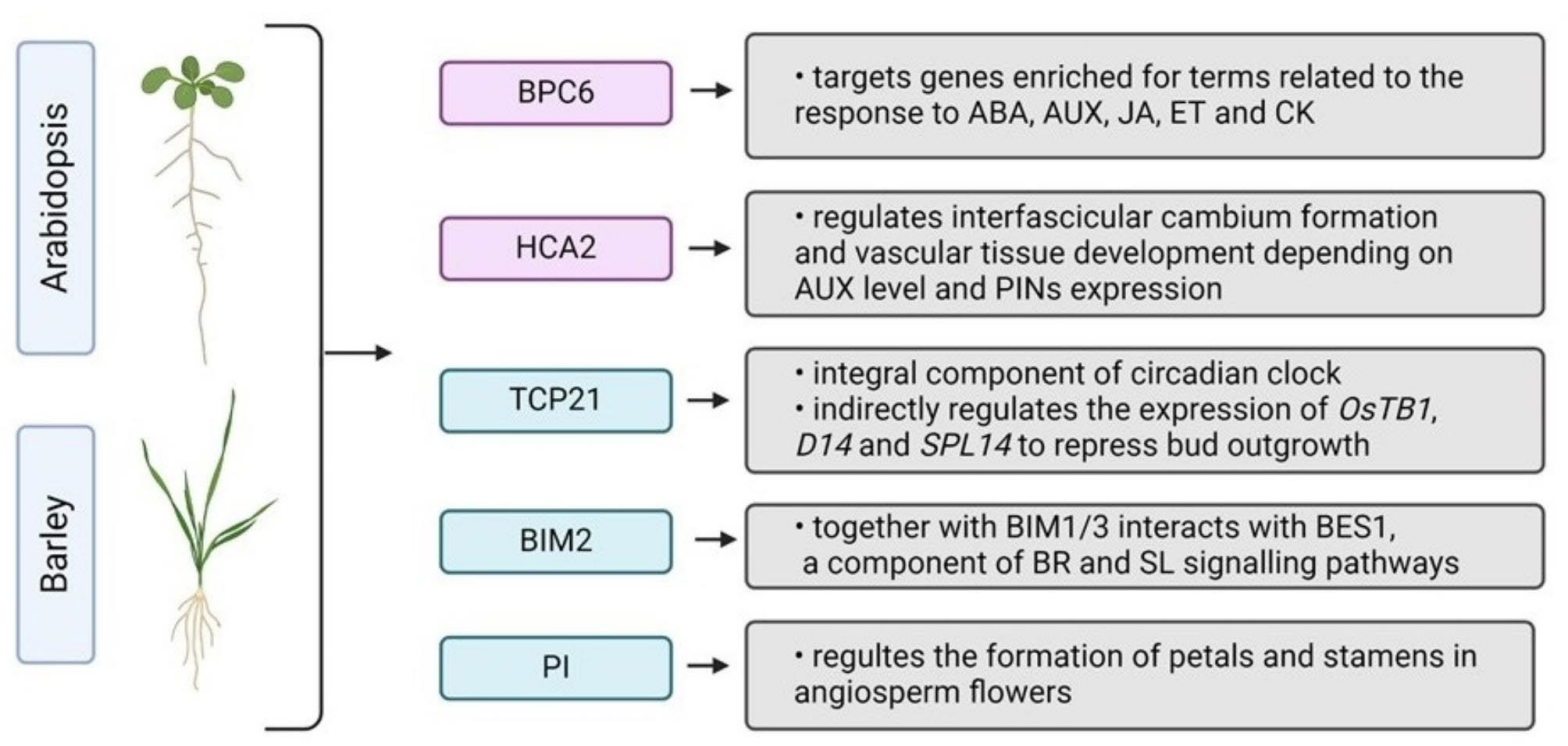
TF with over-represented targets in SL-responsive genes common for barley and Arabidopsis. Pink boxes indicate TF that target genes associated with phytohormonal processes. The illustration was created using BioRender (www.biorender.com).

Finally, we compare all four generated lists of TF that might be involved in SL-signalling that are: i) over-represented TF controlling expression of DEG and genes encoding DAP of 2-week-old *hvd14,* ii) over-represented TF controlling expression of DEG and genes encoding DAP of 4-week-old *hvd14,* iii) over-represented TF controlled expression of hormonal-related DEG/DAP in barley and iv) over-represented TF controlling expression of identified SL-dependent DEG in Arabidopsis (Fig. 4) (Supplementary Data 11). Ultimately, we were able to identify five TF that were common for barley and Arabidopsis in relation to SL-responses, two of which regulate expression of hormone-associated genes/proteins (Fig. 5). Those TF, HORVU7Hr1G012840/AT5G42520 and HORVU6Hr1G069190/AT5G62940, seem to be crucial in the control of SL-dependent processes that are impaired in the *hvd14*, because not only they control the expression of genes that are associated with the observed disbalance of hormonal homeostasis in the mutant, but also they control the expression of the remaining genes whose expression patterns are altered in the SL-insensitive plant. Finally, both TF may have a similar function in Arabidopsis, which means that they may be involved in SL signal transduction and SL cross-talk with other phytohormones in both mono- and dicots.

## Discussion

### SL-insensitivity affects barley shoot architecture

The development of branches increases the number of reproductive structures, such as flowers and fruit-bearing sites, contributing significantly to overall crop productivity^43^. Proper crop branching influences the quantity and quality of the harvest, as it ensures optimal light interception, allowing for more efficient photosynthesis or distribution of nutrients^44^. The primary phytohormone that regulates the plants’ shoot architecture by inhibiting the axillary bud outgrowth is SL^45^. Thus, the SL-insensitive or SL-depleted plants possess more tillers. Our barley mutant *hvd14*, harbouring the mutation in the SL receptor, developed a higher number of tillers than WT (Fig. 1). The differences were most noticeable in 2-month-old plants, with this continuing through the remainder of the plant’s development period, suggesting that this phase of plant growth is the most critical regarding tiller formation. A similar pattern was observed in our previous analysis, where 3-week-old mutant plants produced 50% more tillers than WT, while older plants almost doubled the number of tillers^31^. In hydroponic culture conditions, this changed slightly in favor of *hvd14*, where the SL-insensitive mutant had 60% more tillers compared to WT three-week-old plants^46^. The differences in tiller formation between plants growing in soil or hydroponics conditions might be due to easier access to water and micronutrients. However, the mutation in the gene *HvD14* encoding the SL receptor which has a beneficial effect on the branching level of barley shoots.

### The phenotype of the highly branched SL-insensitive mutant is the result of a fine-tuned network of hormone interactions

Extensive investigation into plant hormone levels and corresponding transcriptional changes in biosynthetic and signalling genes reveals that hormones and corresponding signalling events seem to operate as interconnected networks, with these multi-level and multi-complex hormone interactions affect the vegetative, generative, and plant defence processes in plant life cycle^47^. Thus, mutation in the *HvD14* SL receptor gene prevents SL detection within the plants disturbing the hormone homeostasis of other phytohormones in barley.

The most significant changes in phytohormone content between WT and *hvd14* plants concern ABA and JA, both in the case 2- and 4-week-old barley plants (Fig. 2A,E) (Supplementary Data 2). The lack of functionality of the HvD14 protein contributed to the reduction of ABA levels compared to WT in barley leaves, both in younger and older plants. However, these differences are much more pronounced in older plants, probably related to the different branching level in 2- and 4-week-old mutant plants. Indeed, highly-branched mutants, such as Arabidopsis *max2* and *brc1,* present decreased content of ABA in buds^48^. Partial suppression of branch elongation in these mutants by ABA treatment suggests that ABA may act downstream of SL core signalling pathway. This hypothesis is supported by the fact that *BRC1* expression was not altered after the ABA treatments. Additionally, the ABA biosynthesis mutants *9-cis-epoxycarotenoid dioxygenase 3* (*nced3*) and *aba deficient 2* (*aba2*) exhibited enhanced branching properties, suggesting a potential direct involvement of ABA in the suppression of bud outgrowth. The dissection of Arabidopsis bud into stem, young leaves, young flowers, primary shoot apex and secondary bud tissues showed that ABA accumulates mainly in meristematic tissue, highlighting its role in branching^48^. Indeed, BRC1 binds to and positively regulates the expression of *HOMEOBOX PROTEIN 21* (*HB21*), *HOMEOBOX PROTEIN 40* (*HB40*) and *HOMEOBOX PROTEIN 53* (*HB53*), which together with *BRC1* enhance *NCED3* transcription^49^. It was shown that these three homeobox proteins act in Arabidopsis axillary buds, leading to the ABA accumulation and suppression of bud development. Thus, the highly branched phenotype of our 4-week-old *hvd14* mutant may be caused by impaired cross-talk between SL and ABA pathways.

The second hormone with the most abundant differences in content between WT and *hvd14* plants is JA. The 2-week-old plants showed increased JA content in favour of mutant plants, however this relation changed with the growth and enhanced branching of the mutant (Fig. 2E) (Supplementary Data 2). Four-week-old *hv14* showed reduced JA level compared to WT. JA is known for its involvement in wound healing, plant defence responses and development of flowers. However, recent papers also point to JA involvement in shoot branching. The pear (*Pyrus communis L.)* mutant exhibiting more branched and reduced height phenotype showed significantly higher JA content than parent variety^50^. Additionally, in response to treatment with methyl-JA, the WT phenotype developed fewer branches. A similar situation was observed in identifying LITTLE NINJA (LNJ), a NINJA-related micro-protein that modulates JA signalling by attenuating the repression of JA-signalling^51^. Ectopic expression of *LNJ* in Arabidopsis resulted in dwarf height with branched shoots plants. This effect was transferable between grass species, including barley, rice, and Brachypodium, with the maintenance of high tillering of plants. However, the authors consider this JA-tillering relation to be a consequence of disturbances in the general hormone homeostasis. Hormone profiling of ‘bushy’ transgenic plants revealed altered JA, AUX and CK levels compared to WT. Thus, the altered JA content in our *hvd14* plants might result from cross-talk of JA with other phytohormones.

We also found that mutation in the SL receptor significantly influenced the CK and AUX content (Fig. 2B,C) (Supplementary Data 2). The CK and AUX are considered the main phytohormones that regulate shoot branching^15^. It has been proved that CK promotes bud outgrowths, while AUX acts antagonistically, inhibiting the formation of lateral branches^52^. However, with increasing reports of tillering regulation by SL, we should consider AUX-CK-SL as a critical signalling trio. The *IDEAL PLANT ARCHITECTURE 1* (*IPA1*), also known as *SPL14*, is a direct downstream component of SL-repressor protein in regulating the tiller number in rice^11^. On the other hand, recent research showed that SPL13, a downstream component of SL-signalling, controls CK biosynthesis and affects lateral bud outgrowth^29^. In tomato SL-deficient mutants the expression of *SPL13* is decreased, while the treatment with GR24 results in elevated levels of *SPL13* transcripts. Moreover, knock-out of *SPL13* by CRISPR/Cas9 technique resulted in enhanced growth of lateral buds with higher content of CK and transcripts of *ISOPENTENYL TRANSFERASES 1* (*IPT1*), a CK biosynthesis gene. Additionally, GR24 treatment suppressed CK synthesis and branching of SL-biosynthesis mutants, which was not detected in *spl13* plants. These results demonstrate that *SPL13* acts downstream in SL-signalling pathway to inhibit lateral bud outgrowth by suppression of CK synthesis^29^. It might explain why our barley SL-insensitive and ‘bushy’ mutant presents elevated CK content during development. This hypothesis is supported by the observation that SL and CK act antagonistically on bud outgrowth control, potentially acting on a common target. The treatment of WT pea with GR24 or BA (a synthetic analogue of CK) results in up-regulation or down-regulation of *PsBRC1* gene, respectively, while also affecting plants phenotype^5,6^.

In contrast, AUX content in *hvd14* decreased at the early stages of development compared to WT (Fig. 2B) (Supplementary Data 2). On the other hand, the 4-week-old *hvd14* showed reduced AUX content compared to non-branched younger mutant plants. The SL-AUX model in the regulation of shoot branching assumes that SL regulates the expression of AUX transporters, leading to the increased content of AUX in buds, thus inhibiting its outgrowth^17^. Indeed, in our previous analysis we showed that GR24 treatment of Sebastian plants resulted in increased content of AUX in axillary buds^31^. Analogous observations were noted in the case of different mono- and dicots species^26,53,54^. Additionally, the GR24 treatment resulted in a significant elevation in the amount of AUX in rice nodes and decreased level of *PINs* genes^54^. On the other hand, the NAA treatment reduced the expression of CK biosynthesis genes and increased the expression of *OsD* genes locally in buds, highlighting that CK-AUX-SL cross-talk plays a key role in the regulation of branching. Our highly branched SL-insensitive plants showed altered content of both CK (up-regulation) and AUX (down-regulation), which coincides with the studies presented above and the generally known antagonistic relationships between CK and AUX in the regulation of bud outgrowth.

The last hormone profile affected by the mutation in the *HvD14* gene is SA. SA, similar to JA, is known for its involvement in plant defence responses against biotic and biotic stresses^55^. So far, there is very little research that points to participation of SA in branching. However, in independent research, the increased number of branching was observed in the combination of SA with ascorbic acid^56^ or chelated zinc^57^, in the case of Roselle (*Hibiscus sabdariffa L.)* or sweet pepper (*Capsicum annuum L.*), respectively. On the other hand, the treatment with SA alone of coriander (*Coriandrum sativum*) in field conditions does not affect the number of developing branches^58^. Therefore, due to the lack of direct reports on the involvement of SA in branching, we assume that the altered content of SA in our barley *hvd14* mutant results from disturbed homeostasis of the entire phytohormonal network.

### Transcriptome and proteome changes in *hvd14* correspond with altered hormone homeostasis

Our study, combining transcriptomic and proteomic analyses, revealed various differences contributing to the distinct phenotype between WT and *hvd14* plants. The number of identified DEG was higher in 4-week-old plants, potentially due to more advanced developmental state in addition to the observed phenotype differences in branching. Moreover, only 30 DEG were common to both 2- and 4-week-old plants, indicating that the biological processes occurring in barley plant development are dynamic and development specific (Fig. 3A).

Despite measuring substantial DEG changes that increased with development, we found very comparatively fewer DAPs in either both 2- and 4-week-old plants. This suggests a number of interesting possibilities, including protein turnover, which is not specifically captured by our quantitative proteomic analysis approach, but has been suggested to be a contributing factor to the regularly observed disconnect between a significantly changing transcriptome and an unchanging proteome^59^. Our data highlights this possibility through our measurement of multiple LOXs at both the transcriptomic and proteomic level (Table 1) (Supplementary Data 2 & 3), which in Arabidopsis rosettes been shown to undergo rapid protein turnover^59^. Literature data also indicate the involvement of LOX-like enzymes in the biosynthesis of ABA^60^, being more highly expressed under stressful conditions, so elevated concentrations of plant defence hormones can trigger signal transduction, including SA, JA and ABA, leading to the plant’s response to adverse environmental conditions^36–40^. Here, our 2-week-old plants demonstrated a concurrent and significant transcripts and protein-level changes in two LOX2-like proteins (HORVU6Hr1G000510 & HORVU5Hr1G001180) in WT vs. *hvd14* plants, along with significant protein-level change in LOX1-like protein (HORVU4Hr1G005920). However, by 4-week-old plants, we only still see an up-regulation of transcripts, with no measurable change in LOX2 protein abundance. How these sorts of transcript-protein relationships through events such as protein turnover specifically relates to the developmental differences between WT and *hvd14* plants represents an interesting possibility but is ultimately beyond the scope of this study.

Functional annotation of DEG and DAP showed that almost 15% of identified changes are associated with phytohormone-related processes. Importantly, percentage of individual hormone category, aligns with changes in phytohormone content of *hvd14* (Figs. 2 and 3), showing close relationship between transcriptome/proteome and phytohormonal network. Both ABA and JA showed the most significant changes in 2-week-old, as well as 4-week-old plants (Supplementary Data 2). However, there is limited knowledge about the role of JA in branching, as well as its interactions with SL, we cannot exclude its involvement in the negative regulation of shoot architecture. Thus, the decreased levels of JA in 4-week-old *hvd14* might be linked with more ‘bushy’ phenotype and weaker plant responses to abiotic stress, which was proved in our previous study^32^. On the other hand, the SL and ABA relationship has been widely investigated, especially in terms of signalling pathway cross-talk during plants growth and development, as well as under environmental stress factors^61^. Our previous analysis showed that *hvd14* was insensitive to ABA during germination^62^. Moreover, we proposed that drought-sensitive phenotype of barley SL mutant might be caused by a disturbed ABA metabolism and/or signalling pathways. Thus, the most significant changes in ABA level and expression of ABA-related genes and encoding proteins highlight strong SL-ABA connection. Especially since BRC1 regulates the transcription of ABA-responsive regulators in axillary buds, including *ABA-RESPONSIVE ELEMENET BINDING FACTOR 3* (*ABF3*) and *ABA-INSENTIVIVE 5* (*ABI5*), by binding to the TCP motif present in their promoter sequences^63^.

Our analysis also reveals three LOX genes, that were common between 2- and 4-week-old plants and were associated with phytohormone processes (Table 1). LOX catalyse oxygenation of free polyunsaturated fatty acids into oxylipins, a group of lipid compound, in which JA is included^64^. Literature data also indicate the involvement of LOX-like enzymes in the biosynthesis of ABA via cleavage of carotenoids to produce xanthoxin, which is rate-limiting step in the process^60^. LOX have been shown to be associated with biotic and abiotic stress responses in diverse plant species^65^. Genes encoding LOX are more expressed under stressful conditions, so elevated concentrations of plant defence hormones can trigger signal transduction, including SA, JA and ABA, leading to the plant’s response to adverse environmental conditions^66–70^. However, here we showed that *hvd14* mutant presented lower content of JA, SA and ABA compared to WT, despite the increased expression of the genes encoding LOX during plants growth. Perhaps, the decreased content of these hormones, stimulates their biosynthesis as a feedback regulation, however the accumulation of ABA, JA and SA is blocked by unknown mechanisms. Additionally, the tissue used for transcriptome and proteome analysis was collected from the leaves, while hormone profiling involved the entire above-ground part of the plants.

### SL-dependent TF involved in barley development

**Fig. 6:**
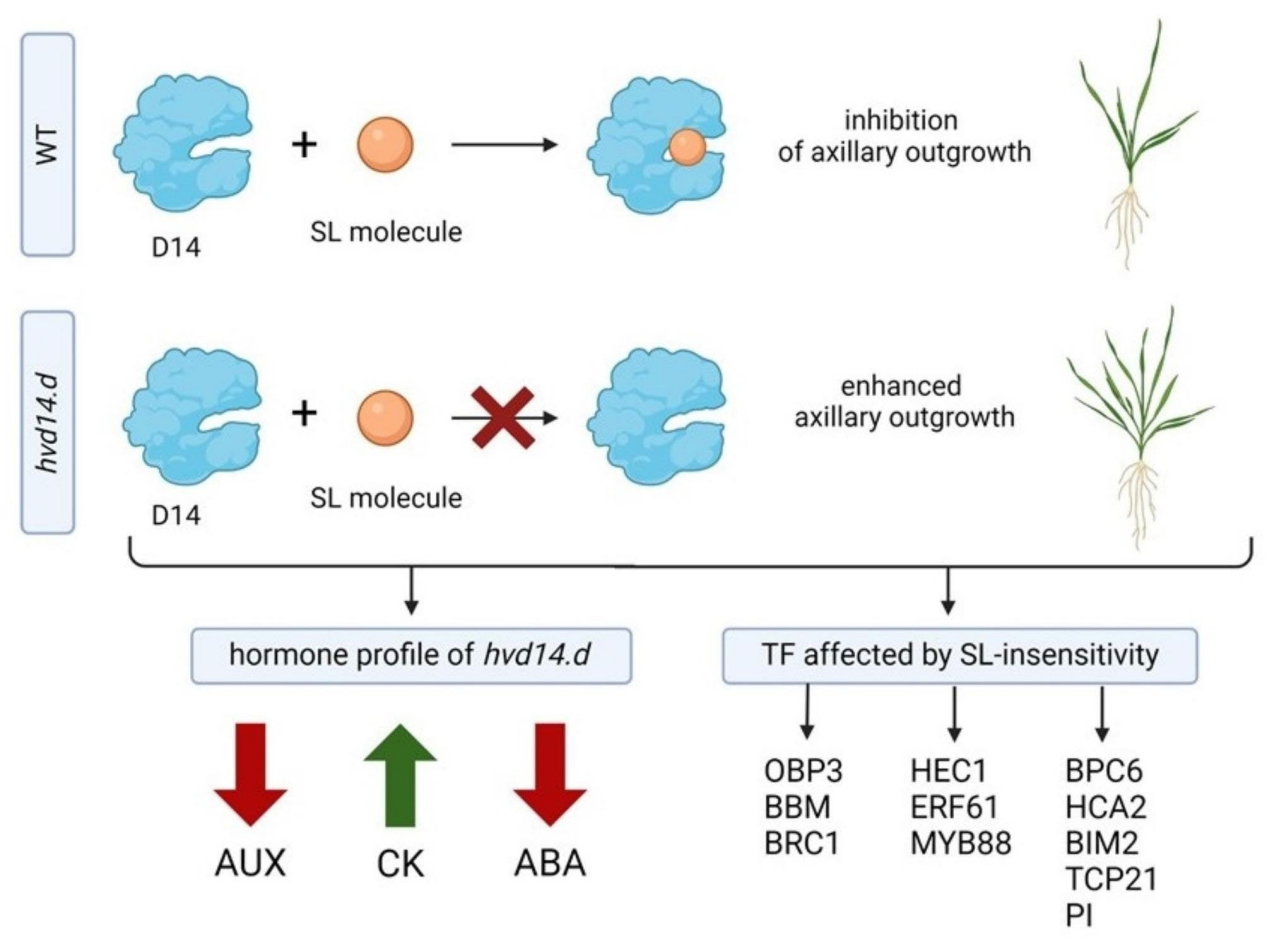
Mutation in *HvD14* gene affects the shoot phenotype of barley due to altered hormone homeostasis and altered TF action. Mutation in *HvD14* gene leads to the loss of SL-molecule binding properties affecting the plants phenotype. Enhanced axillary growth of *hvd14* is connected with altered content of AUX, CK and ABA, as well as changes in TF activity. The illustration was created using BioRender (www.biorender.com).

In well-studied model species like Arabidopsis or rice, the SL signalling pathway and its constituent proteins are extensively documented, from signal perception to repressor degradation. However, our understanding of downstream SL transcriptional responses remains basic. Investigating the transcriptome and proteome of *hvd14* and WT, we identify potential TF influencing SL signal transduction regarding barley development (Fig. 6). In total, 109 potential SL-related TF were identified among DEG and DAP in both 2- and 4-week-old WT and mutant plants, among which four Arabidopsis homologs (*AT3G18550*, *AT5G67060*, *AT1G64380*, *AT2G02820*) were already described as SL-responsive (Supplementary Data 7). One of the genes, *AT3G18550*, encodes a BRC1, the role of which in SL-dependent branching has been extensively demonstrated^3,5,11^. The second gene, *AT5G67060,* encodes HECATE 1 (HEC1) basic helix-loop-helix (bHLH) TF involved in the control of shoot meristem dynamics and gynoecium patterning by modulation of AUX and CK balance^71^. *AT1G64380* encodes an ETHYLENE RESPONSIVE FACTOR 61 (ERF61), which directly regulates the expression of nine genes involved in carotenoid biosynthesis, the precursor of SL or ABA^72^. Thus, the interaction between SL and ABA might occur at the biosynthesis level and be regulated by the feedback loop within SL signalling. Since the SL and ABA cross-talk has been widely documented under control and stress conditions, the ERF61 might be a good candidate for explaining the interaction between these hormones^61^. Another gene, *AT2G02820*, which was differentially expressed in the comparison of 2-week-old *hvd14* and Sebastian plants, encodes MYB DOMAIN PROTEIN 88 (MYB88) involved in a wide range of developmental processes, as well as plants response to abiotic stresses. It was shown that MYB88 and FOUR LIPS (FLP) control the guard cell differentiation and modulation of root architecture under drought conditions. In our previous analysis, we showed that *hvd14* under drought conditions presented a weaker response compared to WT, which was connected with lower leaf relative water content (RWC), impaired photosynthesis, disorganisation of chloroplast structure, slower closure of stomata, as well as altered stomatal density^62^. The impaired SL signalling in *hvd14* mutants could alter the activity of *MYB88*, thereby affecting the phenotype of mutant plants through reduced differentiation of guard cells. Additionally, *MYB88* is directly regulated by BRI1 ETHYLMETHANE SULFONATE SUPRESSOR1 (BES1), described as a co-regulator of Arabidopsis SL repressors.

Next, our bioinformatic approach reveals 33 potentially SL-responsive TF, which may regulate the expression of DEG and genes encoding DAP in 2- and 4-week-old plants (Fig. 4) (Supplementary Data 9). Functional enrichment analysis showed that this set of TF is mainly involved in hormone-associated processes, including response to hormone, hormone-mediated signalling pathways and response to abiotic stresses (Supplementary Fig. 1). These GO terms confirm that SL interacts with different phytohormonal pathways, reflected in disturbed hormone homeostasis in *hvd14* plants. Moreover, SL plays a key role in the activation of plants defence mechanisms under harsh environmental conditions, which might be explained by the alternation of ABA, JA and SA content in *hvd14*, as well as by the annotated function of identified TF.

Furthermore, a reanalysis of data presented by Wang et al*.,* together with our bioinformatical approach, allows us to select 5 genes that encode TF, which may be involved in SL-signalling both in Arabidopsis and barley (Supplementary Data 11). The first one, *AT5G42520,* encodes BASIC PENTACYSTEINE6 (BPC6), which fulfils indispensable functions in plant growth and development by coordinating a complex phytohormone network. BPC6 targets genes enriched for terms related to the response to ABA, AUX, JA, ET and CK, further supporting its role as a regulator of hormone signalling^73^. Indeed, genetic analysis showed that BPC6 promotes lateral root development by regulating ABSCISIC ACID INSENSITIVE 4 (ABI4) expression. Moreover, the roots abnormal phenotype of *bpc1 bpc2 bpc4 bpc6* plants was connected with diminished AUX transport due to reduced PIN1 accumulation, as well as invalid AUX response caused by down-regulation of *PLETHORA 1,2* (*PLT 1,2*) and *AUXIN RESPONSE FACTOR 7* (*ARF7*). The SL interaction between AUX and PINs proteins was already widely described, thus the *BPC6* is a strong candidate that may participate in SL-signalling. The second identified SL-related TF is *AT5G08330* encoding TCP DOMAIN PROTEIN 21 (TCP21). The TCP21 is an integral component of circadian clock, which together with TIMING OF CAB EXPRESSION 1 (TOC1), suppresses the transcription of CIRCADIAN AND CLOCK ASSOCIATED1 (CCA1), a master regulator of plants life cycle^74^. The circadian clock influences diverse developmental processes, especially the shaping of plants architecture^75^. It was shown that rice OsCCA1 positively regulated the expression of OsTB1, D14 and SPL14 to repress bud outgrowth^76^. Moreover, the downregulating and overexpressing OsCCA1 increases and reduces tiller numbers, respectively. Thus, the identified TCP21 might be another player involved in the tillering-circadian clock relation. Another gene, *AT5G62940,* encodes *HIGH CAMBIAL ACTIVITY 2 (HCA2),* which regulates interfascicular cambium formation and vascular tissue development^77^. Secondary growth is mediated by the vascular cambium, a stem cell-like tissue whose proliferating properties are regulated by the AUX and PIN proteins. Additionally, it was shown that SL-deficient mutants display a reduction in secondary growth, and local GR24 treatments stimulate cambium activity^78^. The fourth gene identified as a SL-related TF is *AT1G69010* encoding BES1-INTERACTING MYC-LIKE PROTEIN 2 (BIM2), which together with its homologs BIM1 and BIM3, interacts with BES1 known to activate the expression of BR-induced genes^79^. It was proved that BES1 also participate in SL signalling pathways, regulating the expression of downstream SL-related TF^80,81^, highlighting the possible SL regulation of identified BIM2. The last gene identified as a SL-related TF is *AT5G20240* encoding homeotic protein PISTILLATA (PI), which were already described above.

## Conclusion

A mutation in the *HvD14* gene encoding the receptor protein for SL contributes to semi-dwarf height and an increased number of tillers compared to the parent variety, Sebastian. The regulation of plant branching is influenced by environmental conditions and hormone balance, which affect transcriptomic and proteomic changes. Therefore, our SL-insensitive *hvd14* mutant was subjected to comparative analyses to understand the basis for the altered phenotype of these plants. Profiling the hormone content revealed significant differences in the levels of AUX, CK, and ABA, the role of which is well-known in shaping shoot architecture. It shows that the signaling pathway(s) regulating shoot branching operates as a fine-tuned system requiring a proper balance of hormone content. Moreover, mutation in *HvD14* resulted in a series of DEGs and DAPs, which allowed us to identify strong TF candidates that might be involved in SL signaling. The proposed SL-related TF have been previously indicated to interact with core SL-signaling proteins, as well as proteins primarily involved in AUX transport or ABA signaling, highlighting the complex interplay between these hormonal pathways in regulating plant growth and development. This study provides a comprehensive understanding of the genetic and molecular mechanisms underlying the altered *hvd14* phenotype, offering potential targets for further SL-related research.

## Materials and methods

### Plant material and growth conditions

The *hvd14* mutant carries a homozygous recessive mutation (G725A) in the gene encoding the SL receptor HvD14. This mutant was obtained using chemical mutagenesis after the double treatment of the parent cultivar Sebastian with sodium azide (NaN_3_) and N-methyl-N-nitrosourea^31^. The mutant was double-backcrossed with Sebastian, and grains of both genotypes, Sebastian and *hvd14*, used in the presented studies were collected in this same year (2020).

The 15 grains of WT or mutant genotype, both sourced from the *Hor*TILLUS population^31^ were sown in the boxes (400 × 140 × 175 mm) filled with soil containing a mixture of sandy loam and sand (7:2). Soil was supplied with a nutrient medium (per 1L: 34.3 g NH_4_NO_3_; 40.8 g KH_2_PO_4_; 10 g K_2_SO_4_; 61.5 g MgSO_4_^.^7H_2_O; 0.05 g H_3_BO_3_; 0.03 g CuSO_4_^.^5H_2_O; 0.01 g MnSO_4_^.^H_2_; 0.81 g FeCl_3_^.^6H_2_O) before sowing grains. The plants were grown in a growth chamber under a 16/8 h photoperiod at 20 °C. Analyses were performed on 14- and 28-old-day seedlings.

### Phytohormone measurement

For phytohormone measurement, the whole shoot of seedlings was collected in four biological replicates, each containing four plants. Multiple phytohormone profiling by targeted metabolic analysis was applied to measure phytohormone content in barley tissue, as detailed described previously^82^. Three technical replicates were performed for each of two tissue sets for each genotype and time point. A paired Student’s t-test was applied to check the statistically significant difference between samples.

### Transcriptomic analysis

For RNA-seq analyses, plant tissue (2 cm long fragments of the second leaf located 3 cm below the leaf tip) was collected in four biological replicates, each containing fragments from 4 seedlings of both genotypes. cDNA libraries were prepared following Illumina TruSeq standard procedures and eventually sequenced in an Illumina NovaSeq6000 sequencer, producing 2 × 150 bp paired-end reads. The raw sequencing reads were analyzed using the FastQC software (v0.11.5, Cambridge, UK) to evaluate their quality. Adaptor sequences, empty reads, and low-quality reads (Q < 30 and length < 50 bp) were removed to generate high-quality clean reads. This trimming step was performed with the CLC Genomics Workbench software (v5.0, Qiagen, Vedbæk, Denmark). The clean reads were then aligned and quantified against the barley reference transcriptome using Kallisto (v0.43.0) with default parameters and 100 bootstrap iterations^83^. Differential gene expression analysis was conducted using the DESeq2 package^84^. Genes were considered differentially expressed if they exhibited a log2 fold change of ≥ 1 or ≤ –1 between conditions, with an adjusted *p*-value ≤ 0.01 following Benjamini–Hochberg correction.

### Proteomic analysis

For proteome analysis, the whole shoot of seedlings was collected in four biological replicates, each containing four plants. The tissue was frozen in liquid nitrogen, ground mechanically and then dried using a freeze dryer equipped with a vacuum pump (LAB1ST; FDL1R-1A-220V; Irvine, CA 92,606, USA). The whole procedure requires three critical steps, including protein extraction, trypsin digestion and LC–MS analysis, which were described in detail previously^32^. Briefly, the protein extracts were prepared using an SDS-lysis buffer (4% SDS, 50 mM HEPES–KOH, pH 8.0) and clarified by centrifugation at 20.000 × g for 15 min at room temperature. Protein concentration was determined using a BCA assay (ThermoScientific, 23.225), and 500 µg of protein per sample was reduced with 10 mM DTT at 95 °C for 5 min, cooled, and alkylated with 30 mM iodoacetamide for 30 min in the dark. The reaction was quenched with 10 mM DTT. Samples were then prepared for trypsin digestion using a manual version of the R2-P1 protocol^85^. Peptides (1 µg) were analyzed using an Orbitrap Fusion Lumos Tribrid mass spectrometer, while raw mass-spec files were processed using MaxQuant software version 1.6.14^86^. Spectra were searched against a custom-made decoyed (reversed) version of the barley proteome from the r1 IBSC genome assembly (Phytozome genome ID: 462). Next, using Perseus version 1.6.14.0, reverse hits and contaminants were removed, the data was log-transformed and filtered based on valid quant values in at least 3 of 4 replicates per experimental group. Missing values were imputed from a normal distribution, and differentially abundant proteins were identified using a Benjamini–Hochberg corrected *p*-value threshold of < 0.05^87^.

### Gene ontology enrichment analysis

For GO enrichment analysis, the ShinyGO 0.77 (http://bioinformatics.sdstate.edu/go77/) was used, with FDR cutoff set to 0.05 and the pathway dataset set to GO Biological Process. The tree map of GO Biological Processes were generated with REVIGO (http://revigo.irb.hr/) (the original tree map was modified using a graphic tool), with the resulting list set as medium (0.7) and log_10_ (size).

### Identification of TF

For TF analysis, the protein sequences of DEG and DAP were obtained using the BioMart tool (https://sep2019-plants.ensembl.org/index.html) from the 'Hordeum vulgare genes (IBSC v2)' dataset. Next, with the PlantRegMap (https://plantregmap.gao-lab.org/) tool ‘TF prediction’, the TF among DEG and DAP were identified, parallel with their Arabidopsis homologs.

### Promotor sequences analysis

For promotor sequences analysis, the 1500 bp before the codon START (‘Flank Gene’) of DEG and DAP were downloaded using the BioMart tool (https://sep2019-plants.ensembl.org/index.html) from 'Hordeum vulgare genes (IBSC v2)' dataset. Obtained files were used as input to identify potential regulatory interactions between TF and promoter sequences by PlantRegMap’ Regulatory prediction’ (https://plantregmap.gao-lab.org/), parallel with sorting out the TF which possess over-represented targets in the input gene set.

## Electronic supplementary material

Below is the link to the electronic supplementary material.

Supplementary Material 1

Supplementary Material 2

Supplementary Material 3

Supplementary Material 4

Supplementary Material 5

Supplementary Material 6

Supplementary Material 7

Supplementary Material 8

Supplementary Material 9

Supplementary Material 10

Supplementary Material 11

Supplementary Material 12

## Data Availability

All raw data used in this study can be found in the following repositories. Transcriptomic data: E-MTAB-12804: https://www.ebi.ac.uk/biostudies/arrayexpress/studies/E-MTAB-12804; E-MTAB-12796: https://www.ebi.ac.uk/biostudies/arrayexpress/studies/E-MTAB-12796. Proteomic data: PXD040828: https://www.ebi.ac.uk/pride/archive/projects/PXD040828
