# Supplementary figures and images for: Strigolactone insensitivity affects the hormonal homeostasis in barley
Source: Sci Rep. 2025 Mar 18;15:9375. doi: 10.1038/s41598-025-94430-2 (PMC11920428; doi:10.1038/s41598-025-94430-2)

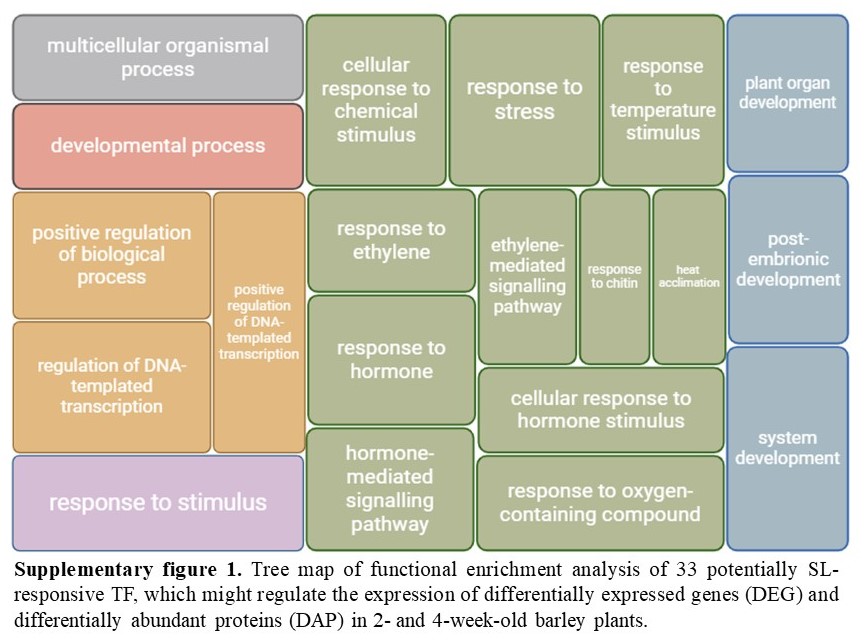

Supplement: Supplementary file 1 — Supplementary Material 1 [file 41598_2025_94430_MOESM1_ESM.jpg]
